# Adipocyte Fatty Acid Binding Protein Potentiates Toxic Lipids-Induced Endoplasmic Reticulum Stress in Macrophages via Inhibition of Janus Kinase 2-dependent Autophagy

**DOI:** 10.1038/srep40657

**Published:** 2017-01-17

**Authors:** Ruby L. C. Hoo, Lingling Shu, Kenneth K. Y. Cheng, Xiaoping Wu, Boya Liao, Donghai Wu, Zhiguang Zhou, Aimin Xu

**Affiliations:** 1 State Key Laboratory of Pharmaceutical Biotechnology, LKS Faculty of Medicine, The University of Hong Kong, Hong Kong, China; 2 Department of Medicine, LKS Faculty of Medicine, The University of Hong Kong, Hong Kong, China; 3 Key laboratory of Regenerative Biology, Guangzhou Institute of Biomedicine and Health, Chinese Academy of Sciences, Guangzhou, China; 4 Department of Geriatrics, Second Xiangya Hospital, Central South University, Changsha, Hunan, China; 5 Department of Pharmacology and Pharmacy, LKS Faculty of Medicine, The University of Hong Kong, Hong Kong, China

## Abstract

Lipotoxicity is implicated in the pathogenesis of obesity-related inflammatory complications by promoting macrophage infiltration and activation. Endoplasmic reticulum (ER) stress and adipocyte fatty acid binding protein (A-FABP) play key roles in obesity and mediate inflammatory activity through similar signaling pathways. However, little is known about their interplay in lipid-induced inflammatory responses. Here, we showed that prolonged treatment of palmitic acid (PA) increased ER stress and expression of A-FABP, which was accompanied by reduced autophagic flux in macrophages. Over-expression of A-FABP impaired PA-induced autophagy associating with enhanced ER stress and pro-inflammatory cytokine production, while genetic ablation or pharmacological inhibition of A-FABP reversed the conditions. PA-induced expression of autophagy-related protein (Atg)7 was attenuated in A-FABP over-expressed macrophages, but was elevated in A-FABP-deficient macrophages. Mechanistically, A-FABP potentiated the effects of PA by inhibition of Janus Kinase (JAK)2 activity, thus diminished PA-induced Atg7 expression contributing to impaired autophagy and further augmentation of ER stress. These findings suggest that A-FABP acts as autophagy inhibitor to instigate toxic lipids-induced ER stress through inhibition of JAK2-dependent autophagy, which in turn triggers inflammatory responses in macrophages. A-FABP-JAK2 axis may represent an important pathological pathway contributing to obesity-related inflammatory diseases.

During obesity, adipocyte dysfunction leads to the elevated circulating free fatty acid (FFA) and its ectopic accumulation in non-adipose tissues induces lipotoxicity by promoting macrophage infiltration and activation, thereby contributes to the development of inflammatory metabolic diseases^1^,^2^. Elevated endoplasmic reticulum (ER) stress is observed in metabolic organs of obese animals^3^. This results in the elicitation of unfolded protein response (UPR) which in turn activates c-Jun N-terminal kinases (JNK) and nuclear factor kappa-light-chain-enhancer of activated B cells (NF-kB) pro-inflammatory signaling pathways^4^ implicating in the molecular mechanism of lipotoxicity. Autophagy is a highly regulated self-degradation process that is essential for cellular survival in response to stress^5^,^6^,^7^. Elevated ER stress induces autophagy via the activation of UPR^8^ to assist the degradation of superfluous proteins that are unable to be eliminated by ER-associated degradation^4^. Emerging evidence show that defective autophagy is associated with various diseases including cancer^9^, neurodegenerative diseases^10^ as well as obesity-related cardio-metabolic diseases^11^,^12^. Autophagy is impaired in the liver of both genetic- and dietary-induced obesity which further promotes ER stress and causes insulin resistance^13^, contributing to the development of non-alcoholic fatty liver disease (NAFLD)^14^. Systemic autophagy insufficiency compromises the adaptation to metabolic stress and promotes the progression from obesity to diabetes^15^. Suppression of autophagy in macrophages by ablating the autophagy-related protein (Atg) 5 promotes cholesterol loading-induced apoptosis and oxidative stress resulting in atherosclerosis^16^. Chronic caloric excess also leads to defective hypothalamic autophagy and induces hypothalamic inflammation with activation of the pro-inflammatory inhibitor of nuclear factor kappa-B kinase subunit beta (IKKβ)/NF-κB pathway hence, promotes the dysregulation of energy and body weight balance in mice^17^. On the contrary, induction of autophagy alleviates ER stress-induced diabetes^18^ and cell death^19^, attenuates progression of atherosclerosis^20^ and reduces steatosis and injury in both alcoholic and non-alcoholic fatty liver diseases^21^. It is also demonstrated that macrophage autophagy is anti-inflammatory and protects against liver fibrosis^22^.

Adipocyte fatty acid binding protein (A-FABP) is a fatty acid chaperone mainly expressed in adipocytes and macrophages^23^. It can be released into the circulation and its serum level is elevated in obese individuals and patients with the metabolic syndrome^24^. A-FABP is a key regulator of inflammatory response in macrophages. It exacerbates lipopolysaccharide (LPS)-induced inflammatory response by forming a finely tuned positive feedback loop with the transcription factor AP-1 and JNK^25^. Toxic-lipids- and LPS- induced productions of inflammatory cytokines are decreased in A-FABP deficient macrophages when compared to wild-type controls^26^. A-FABP is also identified as the mediator of obesity-related complications such as steatohepatitis^27^ and atherosclerosis^26^ by inducing inflammatory activity, inhibiting cholesterol efflux or mediating lipid-induced ER stress in macrophages^28^,^29^.

Since A-FABP, ER stress and autophagy are closely related to obesity and involved in similar pro-inflammatory signaling pathways, the present study aimed to investigate the interplay between A-FABP, ER stress and autophagy in the regulation of toxic lipids-induced inflammatory responses in macrophages. We demonstrated that A-FABP acts as a negative regulator of toxic lipid-induced autophagy by inhibiting the Janus Kinase (JAK) 2 signaling pathway. Impairment of JAK2-dependent autophagy further instigates ER stress, thereby leading to the exaggeration of inflammatory responses in macrophages.

## Results

### Prolonged treatment of palmitic acid induces ER stress and expression of A-FABP but impairs autophagic flux in macrophages

**Figure 1:**
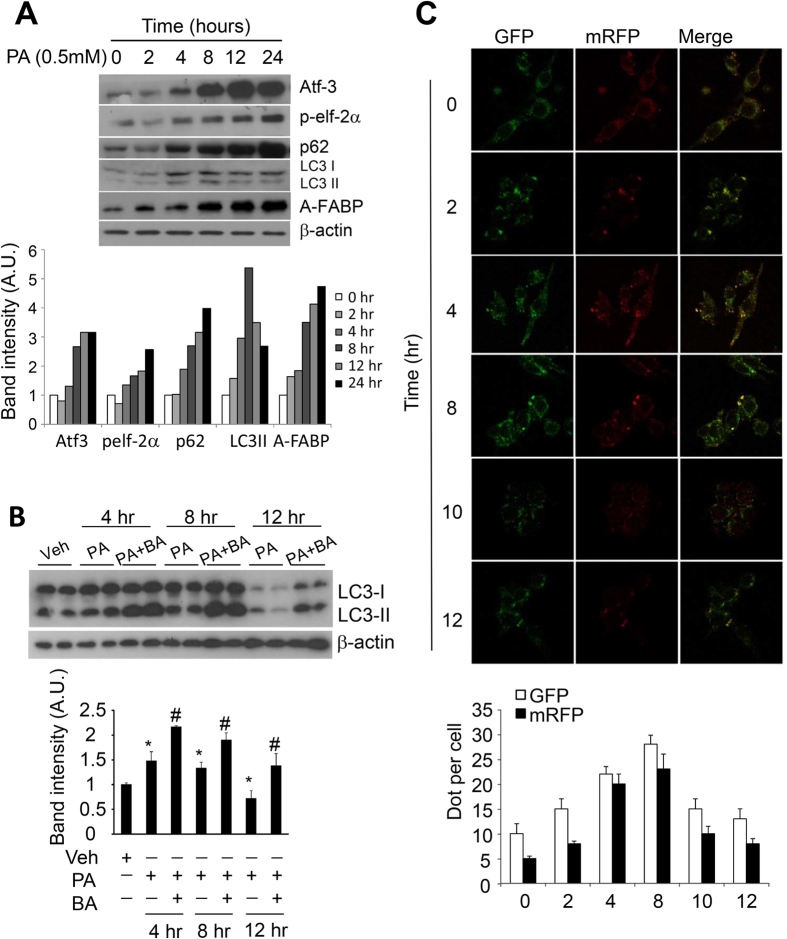
Prolonged treatment of palmitic acid induces ER stress and expression of A-FABP while attenuates autophagy in macrophages. Murine RAW264.7 macrophages were treated with palmitic acid (PA) (0.5 mM) in the presence or absence of autophagy inhibitor bafilomycin A1 (BA; 10 μM) at indicated time points. (**A**,**B**) Cell lysates were subjected to immunoblotting with antibodies of ER stress markers (Atf3, phosphorylated elf-2α (ser 51)), autophagy markers (p62 and LC3I/II), A-FABP and β-actin. The relative expression level of protein was normalized with the expression of β-actin and the densitometric quantification of the immunoblot was shown in the lower panels. (**C**) RAW264.7 macrophages transfected with mRFP-GFP-LC3 construct for 24 hours were treated with PA (0.5 mM) at indicated time points. Representative confocal images were shown and the numbers of GFP and mRFP dots were counted. Values are expressed as means ± S.E.M. *P < 0.05 versus vehicle control; ^#^P < 0.05 versus relative PA-treated macrophages; n = 6.

To elucidate the inter-relationship between A-FABP, ER stress and autophagy, we first examined the effect of the toxic lipid palmitic acid (PA) on ER stress, autophagy and A-FABP expression in macrophages. Treatment of PA increased the expression of the ER stress markers Atf-3 and phosphorylation of elf-2α (Ser51) in RAW264.7 macrophages in a time-dependent manner (Fig. 1A). PA also caused alternative splicing of X-box binding protein (XBP-1) gene (see Supplementary Fig. S1A), suggesting that PA activates the UPR signaling pathways in macrophages. Microtubule-associated protein 1 light chain 3 (LC3) is a well-recognized autophagic marker as cytosolic LC3I is converted to LC3II through lipidation and redistribution to autophagosome membrane in response to autophagic stimuli. The expression level of LC3II directly reflects the number of autophagosomes^30^. Although elevated ER stress is shown to activate autophagy^8^,^19^, the PA-induced elevation of ER stress was associated with a dynamic change of autophagy as the conversion of LC3I to LC3II was markedly increased upon PA induction in the first 8 hours but gradually decreased at later time points which was accompanied by an enhanced accumulation of p62, an ubiquitin-binding scaffold protein that is degraded through autophagy^31^ (Fig. 1A). These data implicated that prolonged treatment of toxic lipid suppresses autophagy. Notably, PA treatment is also associated with increased expression of A-FABP at both mRNA and protein levels in a time-dependent manner (Fig. 1A and see Supplementary Fig. S1B and C).

Recent study demonstrated that the expression of p62 is not always inversely correlated with autophagic activity as it undergoes degradation at the early phase of autophagy but can be restored to basal level at 4 hours due to the compensatory upregulation of its transcription under long term amino acid deficiency^32^. Moreover, the expression of p62 as well as LC3II can be transcriptionally regulated during autophagy^33^,^34^ and it is unclear that whether p62 is solely degraded through autophagy or partially through unbiquitin-proteosome pathway^35^. These may confound the interpretation of p62 and LC3II as autophagic markers. Therefore, measurement of p62 and LC3II in combination with independent experiments was used to validate autophagic flux in this study.

Since the accumulation of LC3II can be due to increased formation or impaired degradation of autophagosome, we verified the effect of PA on autophagic flux by treating macrophages with PA in the presence of bafilomycin A1 (BA) which is an inhibitor of late phase of autophagy^36^. The accumulation of LC3II was significantly increased at 4 hours to 8 hours and was further enhanced when degradation was suppressed by BA indicating the increased autophagic flux. However, sustained PA treatment to 12 hours markedly diminished the accumulation of LC3II (Fig. 1B). Furthermore, the formation of autophagosomes and autolysosomes was monitored by transfecting RAW264.7 macrophages with mRFP-GFP-LC3 construct expressing red (RFP) and green (GFP) fluorescence signals. The yellow signal in the merged image indicated autophagosome while the red signal indicated autolysosome as mRFP fluorescence can be sustained in the acidic condition of lysosomes. Consistent with the result of Western blotting (Fig. 1A), the number of autophagosome and autolysosome was dramatically increased upon PA induction at 4–8 hours while both were reduced after prolonged PA treatment for 10–12 hours (Fig. 1C). These data further confirmed that PA stimulated autophagic flux in macrophages at early stage which was then suppressed after prolonged treatment. Collectively, prolonged treatment of PA increased ER stress while reduced autophagic flux in macrophages which are accompanied by elevated A-FABP expression.

### A-FABP potentiates PA-induced ER stress but inhibits autophagy in macrophages

Defective autophagy promotes ER stress^13^. We next investigated whether or not A-FABP potentiates PA-induced ER stress and regulates autophagy. Since treatment of PA showed an obvious induction of autophagic flux at 8 hours (Fig. 1), we therefore determined the effect of A-FABP on PA-induced autophagy at this time point in the following experiments.

**Figure 2:**
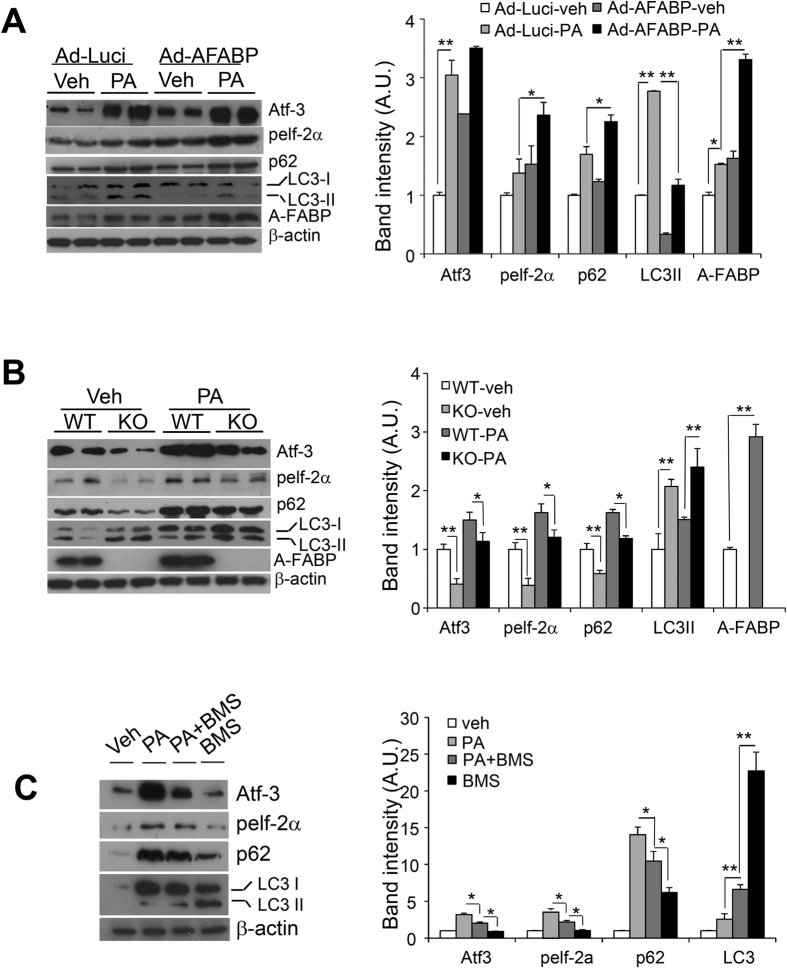
A-FABP potentiates PA-induced ER stress and is accompanied by impaired autophagy. (**A**) RAW264.7 macrophages infected with adenovirus over-expressing either luciferase (Ad-Luci) or A-FABP (Ad-AFABP) for 48 hours or (**B**) primary macrophages isolated from peritoneum of A-FABP KO and wild-type (WT) mice or (**C**) RAW264.7 macrophages pretreated with or without selective A-FABP inhibitor BMS309403 (BMS; 25 μM) for 24 hours were treated with either of Vehicle (Veh; PBS) or PA (0.5 mM) for 8 hours. Cell lysates were subjected to immunoblotting with antibodies as specified. The relative expression levels of proteins were normalized with the expression of β-actin and the densitometric quantification of the immunoblots were shown in the right panels. Values are expressed as means ± S.E.M. *P < 0.05; **P < 0.01; n = 6.

The basal expression of A-FABP was significantly elevated after infection of adenovirus over-expressing A-FABP (Ad-AFABP) when compared with that infected with adenovirus over-expressing luciferase (Ad-Luci). PA induced a significant increase of ER stress and autophagic flux which was accompanied by markedly increased A-FABP expression. Over-expression of A-FABP not only significantly up-regulated the basal ER stress but also exaggerated the PA-induced ER stress in RAW264.7 macrophages (Fig. 2A) which were accompanied by an impaired autophagy with diminished conversion of LC3I to LC3II and increased accumulation of p62 (Fig. 2A) when compared to macrophages with luciferase over-expression.

On the contrary, A-FABP deficient primary macrophages exhibited a significant reduced basal ER stress and accompanied by elevated autophagic flux compared to WT macrophages. The PA-induced ER stress was also markedly ameliorated and was accompanied by enhanced autophagic flux in the A-FABP deficient macrophages (Fig. 2B). Pharmacological inhibition of A-FABP using selective A-FABP inhibitor BMS309403 (BMS) significantly alleviated PA-induced ER stress and enhanced autophagic flux. Treatment with BMS alone also promoted autophagy (Fig. 2C) which was consistent with the data of A-FABP deficient primary macrophages (Fig. 2B). Nevertheless, adenovirus-mediated over-expression of A-FABP further enhanced the mRNA expression levels of ER stress markers such as GRP78, CHOP and spliced XBP-1 and inflammatory cytokines especially MCP-1 and TNF-α upon PA stimulation while treatment with BMS reversed the conditions (see Supplementary Fig. S2A and B). Taken together, these data indicate that A-FABP plays a critical role in modulating PA-induced ER stress and inflammation in macrophages which may via its suppression of autophagy.

### Impairment of autophagy by A-FABP contributes to augmented ER stress

**Figure 3:**
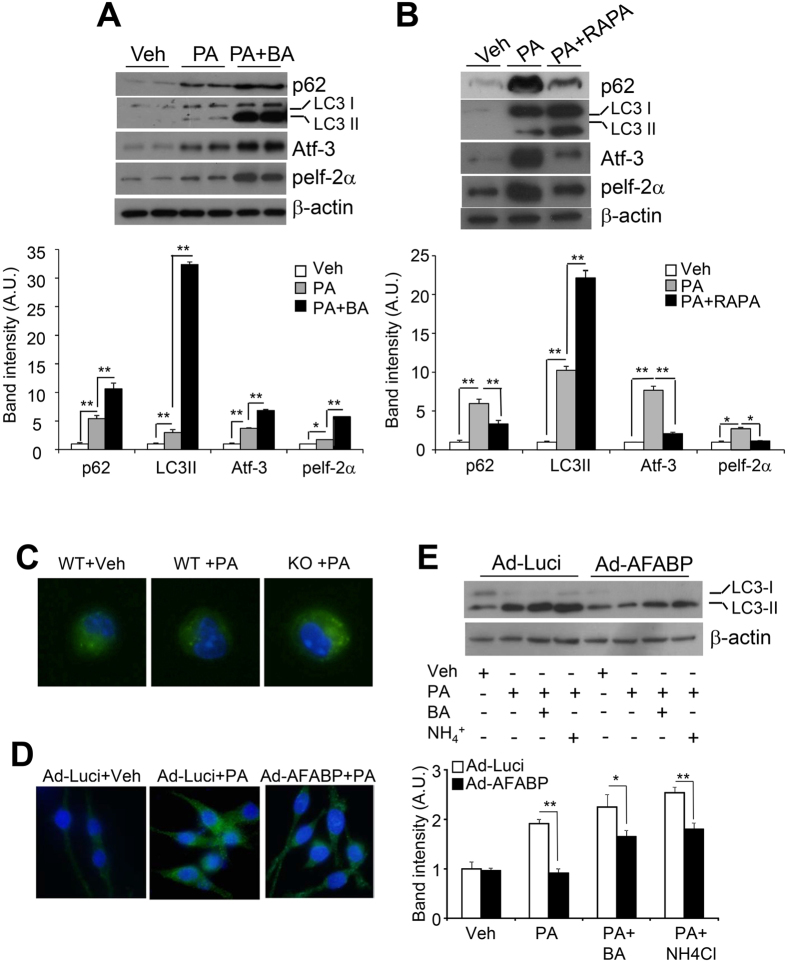
A-FABP inhibits PA-induced autophagy. RAW264.7 macrophages pretreated with (**A**) autophagy inhibitor, Bafilomycin A1 (BA; 10 μM) or (**B**) autophagy inducer, rapamycin (Rapa; 10 μM) for 2 hours were treated with either Veh or PA for 8 hours. Cell lysates were subjected to immunoblotting with antibodies as specified. (**C**) Primary macrophages isolated from the peritoneum of A-FABP KO and WT mice or (**D**) RAW 264.7 macrophages infected with Ad-Luci or Ad-AFABP for 48 hours were treated with either Veh or PA for 8 hours. Immunofluorescence staining of the LC3 puncta structure indicated the formation of autophagosomes and the images were visualized and captured by fluorescent microscope. (**E**) RAW264.7 macrophages infected with Ad-Luci or Ad-AFABP for 48 hours were treated with Veh or PA in the presence or absence of autophagy inhibitor BA (10 μM) or ammonium chloride (NH_4_Cl) (1 μM) for 8 hours. Cell lysates were subjected to immunoblotting. The relative expression levels of proteins were normalized with the expression of β-actin and the densitometric quantification for the immunoblots were shown in the lower panel. Values are expressed as means ± S.E.M. *P < 0.05; **P  < 0.01; n = 6.

To elucidate whether A-FABP instigates PA-induced ER stress via suppression of autophagy, we examined whether or not defective autophagy promotes PA-induced ER stress in macrophages. Suppression of autophagy by treatment of BA greatly abolished PA-induced autophagic flux as indicated by the further increased accumulation of p62. The expression of ER stress markers was also significantly induced (Fig. 3A). On the other hand, PA-induced autophagic flux was apparently enhanced by the treatment of autophagy inducer, rapamycin (RAPA) as indicated by diminished p62 accumulation and was accompanied by alleviated ER stress (Fig. 3B). Likewise, inhibition of autophagy by knocking down the autophagy-related protein Atg7 using silencing RNA approach increased the PA-induced mRNA expression of ER stress markers and inflammatory cytokines (see Supplementary Fig. S3). These data implicate that defective autophagy lead to a further promotion of PA-induced ER stress and inflammation in macrophages.

We next verified the negative regulatory role of A-FABP in autophagy by monitoring the endogenous LC3 puncta formation upon PA treatment in A-FABP deficient- or WT macrophages. Immunofluorescence staining showed that PA-induced formation of LC3 puncta was more pronounced in A-FABP deficient macrophages compared to WT macrophages (Fig. 3C). Conversely, PA-induced formation of LC3 puncta was greatly abolished in RAW264.7 macrophages with adenovirus-mediated over-expression of A-FABP (Fig. 3D). In addition, both basal and PA-induced autophagy (conversion of LC3I to LC3II) and the cumulative LC3II in the presence of late phase inhibitors, BA or ammonium chloride (NH_4_Cl), of autophagy were significantly diminished in macrophages over-expressing A-FABP compared to those of the macrophages over-expressing luciferase (Fig. 3E). These data strongly suggest that elevated A-FABP impairs PA-induced autophagy in macrophages.

### A-FABP alters the expression of autophagy–related protein 7 (Atg7)

**Figure 4:**
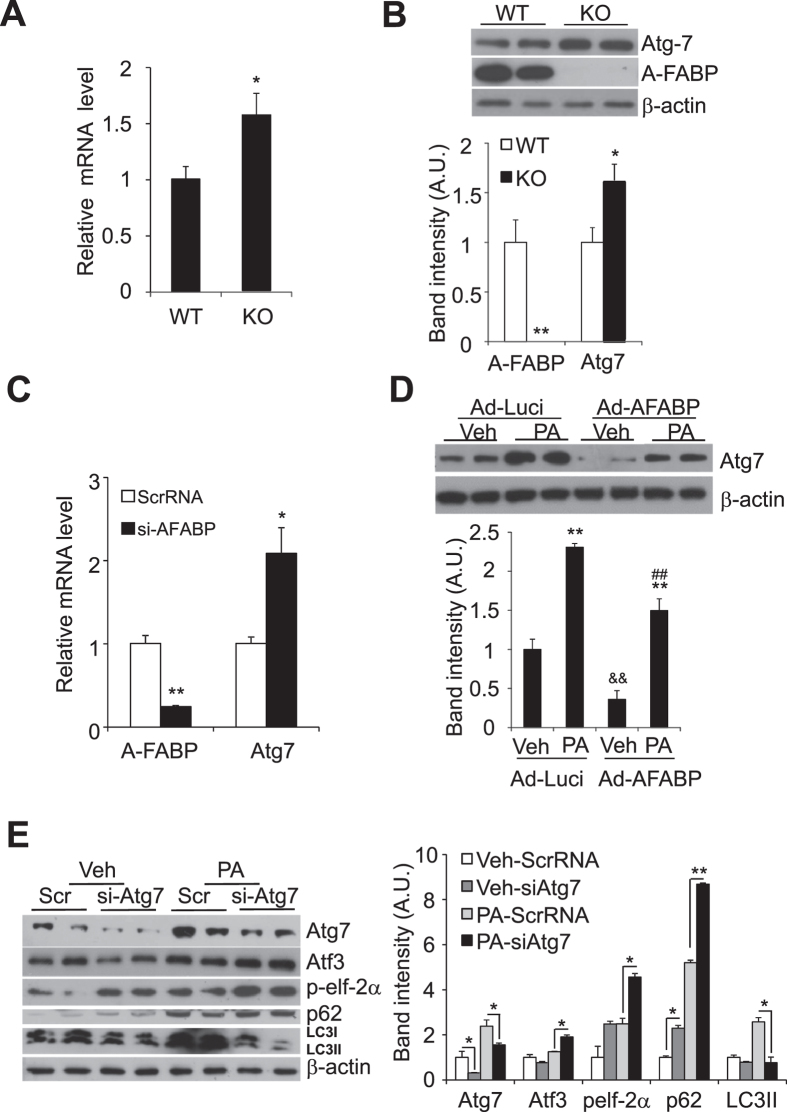
A-FABP modulates the expression of autophagy-related protein (Atg) 7. Peritoneal macrophages were derived from A-FABP KO and WT mice. (**A**) The mRNA abundance and (**B**) protein expression levels of Atg7 and A-FABP were determined by Q-PCR and Western blotting, respectively. RAW264.7 macrophages were transfected with either si-AFABP or ScrRNA (Scr) as control for 48 hours. (**C**) The mRNA abundance of A-FABP and Atg7 were determined by Q-PCR. RAW264.7 macrophages infected with either Ad-AFABP or Ad-Luci as control for 48 hours were treated with Veh or PA for 8 hours. (**D**) Western blot analysis of Atg7. (**E**) RAW264.7 macrophages transfected with si-Atg7 or ScrRNA (Scr) as control for 48 hours were treated with Veh or PA for 8 hours. Cell lysate were subjected to immunoblotting for the expression levels of Atg7, ER stress markers (Atf3, phosphorylated elf-2α (ser 51), autophagy markers (p62 and LC3I/II) and β-actin. The relative expression levels of proteins were normalized with the expression of β-actin. The densitometric quantification of the immunoblot was shown in the lower or right panel. All values are expressed as means ± S.E.M. *P < 0.05; **P < 0.01 vs relative control; ^&&^P < 0.01, Ad-Luci vs Ad-AFABP; ^##^P < 0.01 vs relative control; n = 6.

We then elucidated how A-FABP contributes to defective autophagy. Our data showed that both mRNA and protein levels of autophagic essential protein Atg7 were significantly increased in A-FABP deficient macrophages when compared to WT macrophages (Fig. 4A and B). Knocking down of A-FABP also induced the mRNA expression of Atg7 in RAW264.7 macrophages (Fig. 4C). Conversely, over-expression of A-FABP significantly diminished both basal and PA-induced Atg7 expression (Fig. 4D). We further evaluated the effect of inhibition of Atg7 on PA-induced autophagy and ER stress. As expected, knockdown of Atg7 significantly attenuated PA-induced autophagic flux as indicated by reduced LC3 II and increased accumulation of p62 comparing to the relative controls. This impaired autophagic flux was also accompanied by increased expression of ER stress markers (Fig. 4E). These data implicate that the presence of A-FABP reduces the expression of Atg7 contributing to defective autophagy thus increases ER stress in macrophages.

### PA-induced autophagy is JAK2-dependent, Inhibition of JAK2 signaling potentiates ER stress

**Figure 5:**
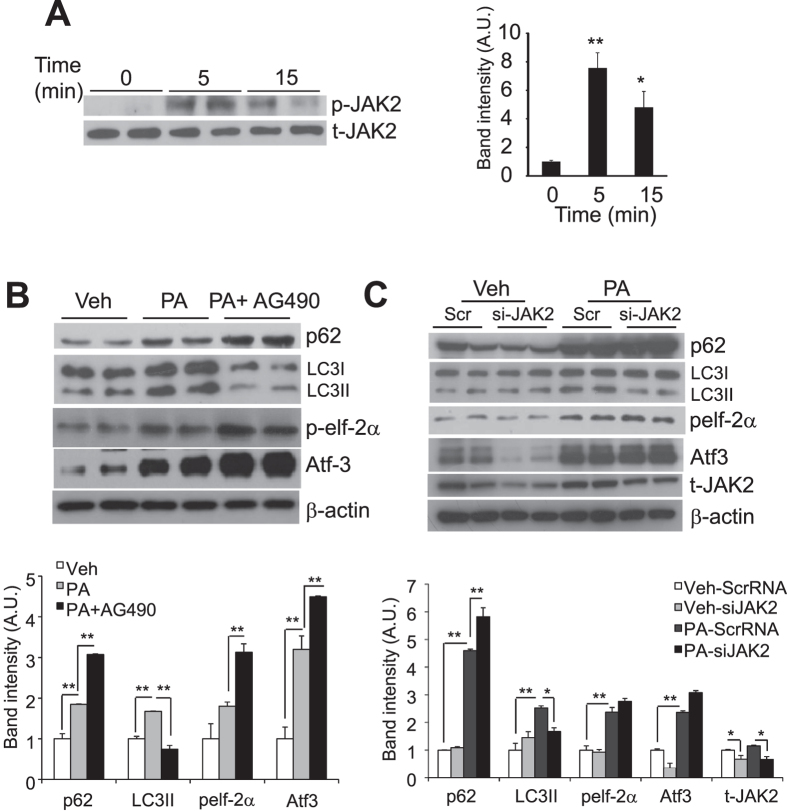
PA-induced autophagy is JAK2-dependent and inhibition of JAK2 signaling enhances PA-induced ER stress. (**A**) RAW264.7 macrophages were treated with PA (0.5 mM) at the indicated time points. (**B**) RAW264.7 macrophages pre-treated with or without JAK2 specific inhibitor AG490 (25 μM) for 2 hours or (**C**) RAW 264.7 macrophages transfected with ScrRNA (Scr) or si-JAK2 for 48 hours were stimulated with Veh or PA for 8 hours. In all experiments, cell lysates were subjected to immunoblotting with antibodies as indicated. The relative expression levels of proteins were normalized with the expression of β-actin or total JAK2 (t-JAK2) and the densitometric quantification of the immunoblots were shown in the right or lower panel. All values are expressed as means ± S.E.M. *P < 0.05; **P < 0.01; n = 6.

JAK2 was shown to mediate autophagy in hepatoma and U87 glioblastoma cells^37^. FFA-bound A-FABP also interacts and attenuates JAK2 activity^38^. To further investigate the underlying mechanism whereby A-FABP impairs PA-induced autophagy, we first examined whether JAK2 is involved. Treatment of PA induced a rapid and significant increase in the phosphorylation of JAK2 (Tyr1007/1008) which was then decreased after 15 minutes stimulation in RAW264.7 macrophages (Fig. 5A). The effect of JAK2 on autophagy was elucidated using its specific inhibitor AG490. ER stress was markedly induced after PA stimulation for 8 hours and was accompanied by elevated autophagic flux. However, treatment of AG490 significantly abolished the PA-induced autophagic flux associating with a further induction of ER stress (Fig. 5B). Meanwhile, suppression of JAK2 signaling using silencing RNA specific for JAK2 (si-JAK2) increased PA-induced ER stress and was accompanied by defective autophagy (Fig. 5C). All these data suggest that activation of JAK2 is indispensable for PA-induced autophagy while its suppression further promotes ER stress.

### A-FABP suppresses PA-induced JAK2 activation which may associate with reduced expression of Atg7. 

**Figure 6:**
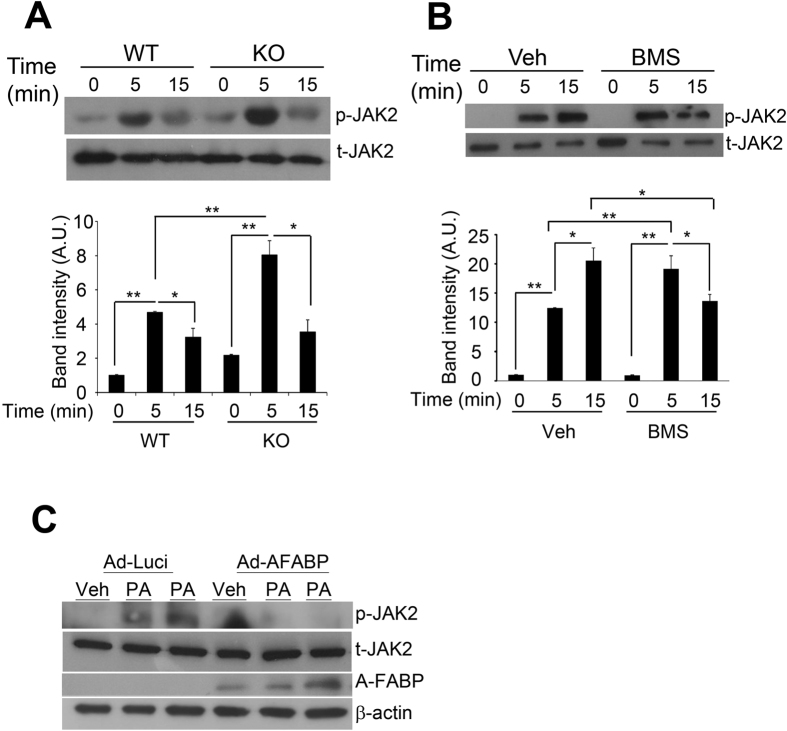
A-FABP attenuates PA-induced JAK2 activation. (**A**) Peritoneal primary macrophages of WT and A-FABP KO mice or (**B**) RAW 264.7 macrophages pretreated with or without BMS (25 μM) for 24 hours were treated with Veh or PA at the indicated time points. (**C**) Primary macrophages of A-FABP KO mice infected with either Ad-Luci or Ad-AFABP for 48 hrs were treated with Veh or PA for 5 mins. Cell lysates were subjected to immunoblotting with the antibodies specified. The relative expression levels of proteins were normalized with the expression of β-actin or total JAK2 (t-JAK2) and the densitometric quantification of the immunoblots were shown in the lower panels. All values are expressed as means ± S.E.M. *P < 0.05; **P < 0.01; n = 6.

**Figure 7:**
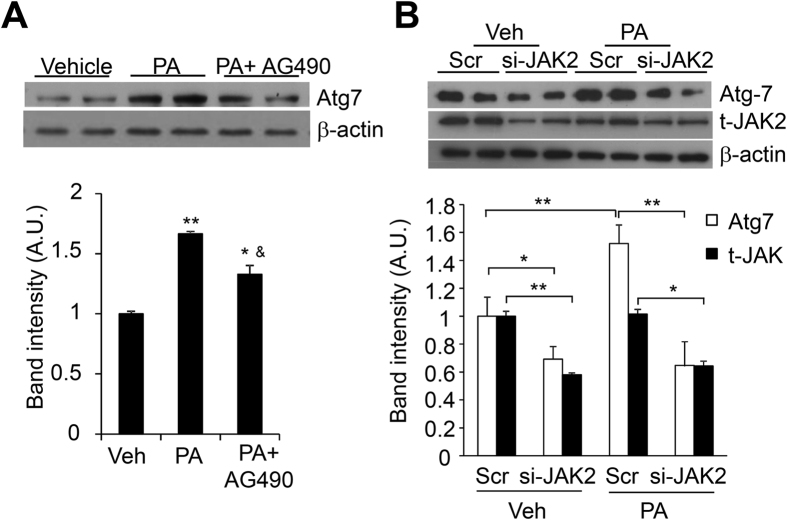
Suppression of JAK2 activity reduces the expression of Atg7. RAW264.7 macrophages (**A**) pre-treated with AG490 (25 μM) for 2 hours or (**B**) transfected with si-JAK2 or ScrRNA (Scr) as control for 48 hours were treated with Veh or PA for 8 hours. Cell lysate were subjected to immunoblotting for the expression levels of Atg7, total JAK2 and β-actin. The relative expression levels of proteins were normalized with the expression of β-actin. The densitometric quantification of the immunoblot was shown in the lower panels. All values are expressed as means ± S.E.M. *P < 0.05, **P < 0.01 vs relative control; ^&^P < 0.05 vs vehicle control; n = 6.

We next investigated whether A-FABP inhibits PA-induced autophagy through its suppression on JAK2 activation. JAK2 activity was markedly induced in both WT and A-FABP KO primary macrophages upon PA treatment while the extent of JAK2 phosphorylation (Tyr1007/1008) was more apparent in A-FABP deficient macrophages than that of WT macrophages (Fig. 6A). Similar result was observed in RAW264.7 macrophages pretreated with BMS followed by PA stimulation (Fig. 6B). Nevertheless, over-expression of A-FABP significantly abolished PA-induced activation of JAK2 in A-FABP deficient macrophages which further ensured the effect of A-FABP on attenuating JAK2 signaling (Fig. 6C). Furthermore, inhibition of JAK2 signaling by treatment of AG490 reduced PA-induced Atg7 expression (Fig. 7A). Both basal and PA-induced Atg7 expression was also dramatically decreased in RAW macrophages when JAK2 was knocked down (Fig. 7B). These data suggest that A-FABP suppresses JAK2 activation which may in turn reduce the expression of Atg7 leading to defective autophagy.

### A-FABP-mediated impairment of autophagic flux attenuates phagocytosis and promotes M1 macrophage polarization

**Figure 8:**
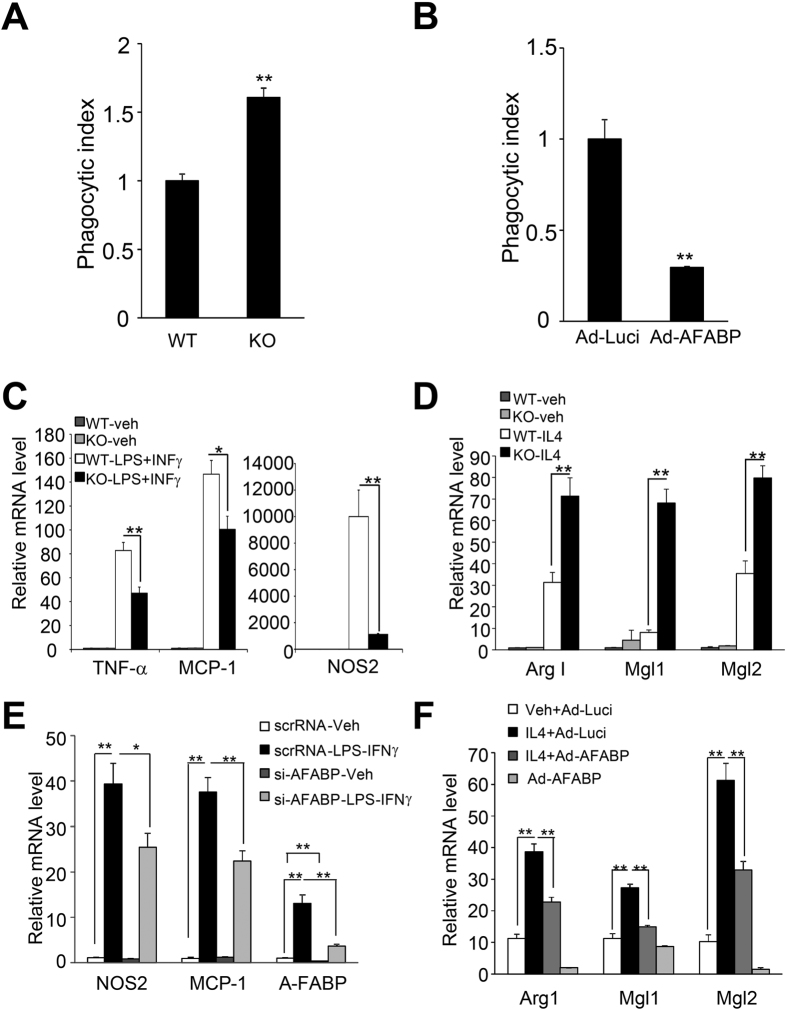
A-FABP attenuates phagocytic activity and M2 macrophage polarization but enhances M1 macrophage polarization. (**A**) WT and A-FABP deficient macrophages or (**B**) A-FABP deficient macrophages infected with adenovirus-over-expressing luciferase or A-FABP were treated 1:500 dilution of latex beads-rabbit IgG-FITC complex or no beads for 4 hours. Phagocytosis of FITC-labeled latex beads by macrophages was measured by flow cytometry. The phagocytosis index is expressed as fold change. (**C**,**D**) Peritoneal macrophages from WT and A-FABP KO mice were treated with vehicle (Veh) and (**C**) LPS (10 ng/ml) and IFNγ (100 ng/ml) or (**D**) IL4 (10 ng/ml) for 8 hours. (**E**,**F**) RAW 264.7 macrophages were transfected with either (**E**) siA-FABP or ScrRNA for 48 hours followed by treatment with LPS (10 ng/ml) and INF-γ (100 ng/ml) for 8 hours or (**F**) infected with either Ad-AFABP or Ad-Luci as control for 48 hours followed by treatment with IL-4 (10 ng/ml) for 8 hours. The mRNA abundance of M1 polarization markers (TNF-α, MCP-1 and NO2) and M2 polarization markers (Arg I, Mgl1 and Mgl2) were determined by Q-PCR. All values are expressed as means ± S.E.M. *P < 0.05, **P < 0.01; n = 4.

We further evaluated the potential implication of impaired autophagic flux regulated by A-FABP in macrophages. The phagocytic activity of A-FABP deficient macrophages was significantly higher than that of the WT macrophages (Fig. 8A). On the contrary, adenovirus-mediated over-expression of A-FABP greatly suppressed the phagocytic activity of A-FABP deficient macrophages (Fig. 8B). Comparing to WT macrophages, LPS-INFγ-induced expression of M1 macrophage polarization markers was significantly diminished while the expression of IL4-induced M2 markers was significantly enhanced in A-FABP deficient macrophages (Fig. 8C,D). Similarly, knockdown of A-FABP in RAW 264.7 macrophages markedly alleviated LPS-INFγ induced M1 macrophage polarization while over-expression of A-FABP significantly attenuated the IL4-induced M2 macrophage polarization (Fig. 8E,F). Taken together, these data imply that defective macrophage autophagy caused by toxic-lipid induced elevation of A-FABP may lead to impaired phagocytosis and increased M1 macrophage polarization which may further contribute to the development of inflammatory diseases such as atherosclerosis^39^ and liver injury^40^.

## Discussion

**Figure 9:**
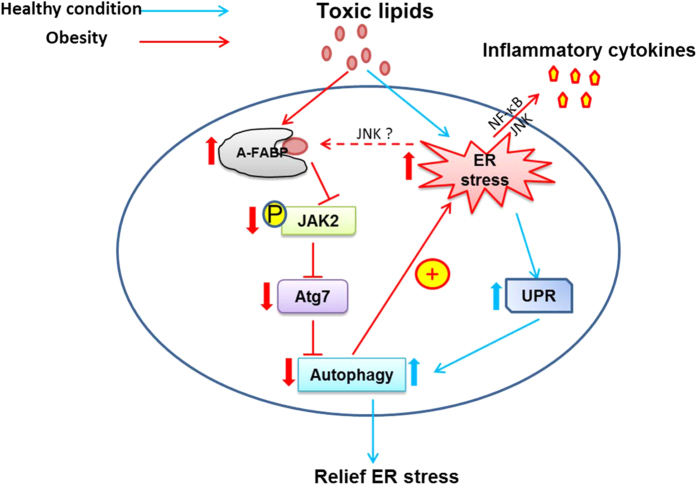
Schematic diagram illustrating the underlying mechanism whereby A-FABP regulates toxic lipid-induced ER stress and inflammation in macrophages. Under healthy condition (indicated by blue arrow), exposure to toxic lipids activates the UPR leading to the induction of autophagy to relief ER stress. Under obese condition (indicated by red arrow), prolonged exposure to toxic lipids increases the expression of A-FABP. FFA-bound A-FABP interacts and attenuates JAK2 activity leading to a reduced expression of Atg7 contributing to defective autophagy. Impaired autophagy further promotes ER stress which in turn activates pro-inflammatory signaling pathways and increases the production of inflammatory cytokines. This eventually contributes to the development of obesity-related inflammatory diseases.

Although impaired autophagy in macrophages has mild effects on the metabolic function and adipose tissue inflammation of obese mice^40^,^41^, defective autophagy has been shown to underlie the pathogenesis of obesity-related disorders such as steatohepatitis, atherosclerosis and cardiomyopathy associating with increased ER stress and inflammation^13^,^16^,^40^,^42^. A-FABP is a key player in chronic inflammation in obesity. It mediates high fat diet induced steatohepatitis by modulating inflammatory response in hepatic resident macrophage Kupffer cells^27^. A-FABP also potentiates lipid-evoked inflammation in macrophages through altering the lipid composition resulting in elevated ER stress and contributes to atherosclerosis^29^. Here, we provide the first evidence that A-FABP can act as an autophagy inhibitor by attenuating JAK2 activation in response to toxic lipid stimulation to instigate ER stress which subsequently leads to the exaggeration of inflammatory activity in macrophages (Fig. 9).

In the present study, prolonged treatment of macrophages with toxic lipid palmitic acid (PA) was performed to mimic lipotoxicity under obese condition to dissect the inter-relationship of A-FABP, ER stress and autophagy in the regulation of macrophage inflammation. PA is shown to induce autophagy via different signaling pathways including JNK2 in pancreatic β cells^43^, and protein kinase C in mouse embryonic fibroblasts or hepato-carcinoma HepG2 cells^44^. JAK is a family of non-receptor tyrosine kinase. Though JAK is well-recognized to mediate signals of cytokines and growth factors via JAK-STAT pathway^45^, its activation induces autophagy in hepatoma^46^ and glioblastoma cells^47^ by increasing the expression of autophagy inducer BCL2/adenovirus E1B 19kDa interacting protein 3 (BNIP3)^37^. Consistently, we showed that JAK2 signaling is essential for PA-induced autophagy in macrophages which may via increasing the expression of autophagy-related protein Atg7. In addition, activation of JAK2-STAT3 signaling pathway alleviates myocardial ischemic reperfusion induced ER stress in heart^48^. Activation of JAK2-STAT3 signaling by stimulating nicotinic acetylcholine receptors attenuates inflammatory activity in intestinal macrophages^49^. Collectively, these data support our findings that attenuation of JAK2 signaling exaggerates PA-induced ER stress and inflammation.

Prolonged PA treatment is shown to attenuate autophagic flux in pancreatic β cells^50^ and cardiomyocytes^51^ resulting in cell apoptosis. Consistently, we showed that PA induces ER stress and autophagy at early stage while autophagy is gradually impaired due to the elevated expression of A-FABP and accompanied by a further enhanced ER stress and inflammation in macrophages after prolonged PA treatment. A-FABP can physically interact with, hormone sensitive lipase (HSL) and coactivator of adipose triglyceride lipase (ATGL), comparative gene identification-58 (CGI-58) to mediate lipolysis and lipid signaling^52^,^53^, and peroxisome proliferator-activated receptor gamma (PPAR-γ) to regulate gene transcription^54^. A-FABP also promotes ubiquitination and degradation of PPAR-γ via direct interaction of the two proteins^55^. In line with previous findings showing that FFA-bound A-FABP binds with basal unphosphorylated JAK2 and suppresses its activity^38^, we showed that elevated A-FABP attenuates JAK2 signaling in response to toxic lipid stimulation leading to impaired autophagy. On the contrary, treatment of A-FABP specific inhibitor BMS309403 (BMS) which competes the fatty acid binding site of A-FABP potentiates PA-induced JAK2 phosphorylation suggesting that the suppressive effect of A-FABP on JAK2 signaling is FFA-bound dependent. These data indicate that A-FABP is a FFA sensor modulating cellular autophagy probably through protein-protein interaction. Notably, treatment of BMS alone also promotes autophagic flux but whether it works through other mechanisms to enhance autophagy warrants further investigations.

Elevated A-FABP in response to toxic lipid stimulation down-regulates JAK2 activity which is associated with reduced expression of autophagic essential protein Atg7. Atg7 is the ubiquitin-E1-like enzyme responsible for autophagosome formation by mediating the conjugation of LC3I to the membrane lipid phosphatidylethanolamine for LC3II formation^56^. It is essential for both basal and stimulated autophagy. Homozygous knockout of Atg7 is neonatal lethal^57^. Crossing global Atg7 haploinsufficency mice with ob/ob mice was shown to impair the adaptation to increased metabolic load and inflammation^15^. Organ-specific Atg7 knockout mouse are used as autophagy defective model to study the role of autophagy in different diseases^58^,^59^. Notably, in genetic and dietary models of obesity, defective hepatic autophagy is observed and is accompanied by severely reduced expression of Atg7 contributing to elevated ER stress^13^. In line with our data, Atg7 deficient T lymphocytes show increased ER stress^60^. Macrophages from Atg7 deficient mice also exhibit elevated inflammatory response^61^,^62^. These findings support our conclusion that attenuation of autophagy by elevated A-FABP in response to PA stimulation contributes to increased ER stress and exaggerated inflammatory response. Recent studies show that heat shock factor 1 and cAMP response element-binding protein (CREB) regulate the expression of Atg7 by direct binding to Atg7 promoter in breast cancer cells^63^ and hepatocytes^64^, respectively. By searching online database, a STAT1/3 binding site is located on Atg7 promoter implicating that PA-induced Atg7 expression may through the activation of JAK2-STAT 1 or 3 pathways. This further supports attenuation of Atg7 expression by A-FABP through its interaction with JAK2.

In the present study, we show that lipotoxicity induces the expression of A-FABP leading to defective macrophage autophagy which eventually potentiates ER stress and inflammatory activity. Over-expression of A-FABP in macrophages also impaired phagocytic activity and enhanced M1 macrophage polarization. Previous studies showed that defective phagocytic clearance of apoptotic cells promotes plaque necrosis in advanced atherosclerosis^39^ while macrophage autophagy confers protective effect in advanced atherosclerosis by attenuating plaque necrosis, macrophage apoptosis and oxidative stress^16^. Autophagy also regulates cholesterol efflux from macrophage foam cells^65^. Furthermore, impaired autophagy in Kupffer cell enhances immune response in diet-induced obese mice by promoting M1 macrophage polarization leading to hepatic inflammation and progression to liver injury^40^. Macrophage A-FABP is implicated in the pathogenesis of atherosclerosis by inducing inflammatory cytokine production and cholesterol esters accumulation^26^. A-FABP is also an important contributor to both LPS-induced acute liver injury and diet-induced steatohepatitis by potentiating inflammation in Kupffer cells^27^. Collectively, these data suggest that inhibitory effect of A-FABP on macrophage autophagy may underlie the development of atherosclerosis and steatohepatitis.

Our previous study showed that A-FABP mediates LPS-induced inflammatory responses by forming a positive feedback loop with JNK and AP-1. LPS induces transactivation of A-FABP through the activation of JNK/AP-1 signaling pathway while elevated A-FABP further potentiates JNK/AP-1 action^25^. As JNK is the downstream target of ER stress, it is plausible that increased ER stress after prolonged exposure to toxic lipids can further enhance the expression of A-FABP which inactivates JAK2 signaling pathways contributing to harmful vicious cycle.

In summary, the present findings demonstrated a nonconventional role of A-FABP in the regulation of autophagy which potentiates toxic lipids-induced inflammation in macrophages by instigating ER stress through inhibition of the JAK2-dependent autophagy. As elevated expression of macrophage A-FABP is observed in inflammatory diseases associating with obesity^26^,^27^ and its suppressive effect on autophagy is FFA-bound dependent, A-FABP may be a central regulator in autophagy during obesity contributing to the development of obesity-related inflammatory disease. Furthermore, ER stress and inflammation regulate and promote each other during obesity^66^ and A-FABP is involved in both of them, inhibition of A-FABP and induction of autophagic flux may be the potential therapeutic strategies for the treatment of obesity-related inflammatory complications.

## Methods

All the following experimental protocols were approved and carried out in accordance to the guidelines by The University of Hong Kong. All the experimental protocols related to animals were approved by and carried out in accordance to the guidelines from the Committee on the Use of Live Animals in Teaching and Research at the University of Hong Kong.

### Chemicals and Reagents

Palmitic acid (PA), bafilomycin A1 (BA), ammonium chloride (NH_4_Cl), JAK2 specific inhibitor AG490, rapamycin (RAPA) and free fatty acid (FFA) free-bovine serum albumin (BSA) and lipopolysaccaride (LPS) were purchased from Sigma Aldrich (St. Louis, Missouri, USA). Thioglycollate was from Difco (Detroit, MI, USA). Interferon γ (INF-γ) and interleukin-4 (IL-4) were purchased from R&D systems. All the antibodies used in the experiments were purchased from Cell signaling Technology Inc. (Danvers, MA, USA), R&D Systems (Minneapolis, MN, USA) or Santa Cruz Biotechnology Inc. (Dallas, Texas, U.S.A.). BMS309403 was synthesized as previously described^67^.

## Animals

### Generation of A-FABP deficient mice

The A-FABP KO mice were generated as previously described^68^. A-FABP KO mice were crossed into C57BL/6N background for nine generations before the experiments. All mice were housed in a temperature-controlled facility (23 °C ± 1 °C) with a 12-hour light/dark cycle, and permitted free access to water and standard mouse chow (Purina, Framingham, MA, USA) and water. All experimental protocols were approved by the Committee on the Use of Live Animals in Teaching and Research at the University of Hong Kong.

### Generation of adenovirus over-expressing A-FABP

Adenoviruses over-expressing A-FABP (Ad-AFABP) was generated according to the manufacturer manual of AdEasy^TM^ XL Adenoviral Vector System (Stratagene, La Jolla, California, USA) as previously described. Recombinant adenovirus encoding luciferase (Ad-Luci) was described previously^69^. Both recombinant adenoviruses were purified according to the manufacturer manual of AdEasy Virus Purification Kit (Stratagene).

### Cell culture

Murine RAW264.7 macrophages were maintained in DMEM supplement with 10% Fetal Bovine serum, 50 U/ml penicillin G and 50 U/ml streptomycin sulfate (Invitrogen, Carlsbad, CA, USA). Peritoneal macrophages were isolated from A-FABP KO mice or their wild-type (WT) littermates 3 days after intraperitoneal injection of 4% thioglycollate. Cells were treated with FFA-free BSA supplemented media with or without palmitic acid (0.5 mM) in the presence or absence of various autophagy inhibitors, inducer or JAK2 inhibitor AG490 as indicated in the figures. In some cases, RAW264.7 macrophages were transfected with scramble RNA or silencing RNAs targeting A-FABP (si-AFABP), Atg7 (si-Atg7; Ambion), JAK2 (si-JAK2) or scramble RNA (ScrRNA) using transfection reagent lipofectamine 2000 (Invitrogen) or infected with adenoviruses over-expressing A-FABP (Ad-AFABP) or luciferase (Ad-Luci) as control of fifty multiplicity of infection (M.O.I.) for 48 hours followed by treatment with the above chemicals. The sequences of the silencing RNA are listed in the Supplementary Table 1.

### Determination of XBP-1 splicing by RT-PCR

The X-box binding protein-1 (XBP-1) mRNA splicing in macrophages was analysed using PCR as described previously^70^. The sequences of primers used for PCR amplification of mouse XBP-1 are listed in Supplementary Table S1 (see Supplementary Table S1). The PCR products were resolved by electrophoresis on a 5% polyacrylamide gel and visualized by ethidium bromide staining.

### Determination of palmitic acid (PA)-induced autophagic flux in macrophages

RAW264.7 macrophages were transfected with GFP-mRFP-LC3 construct (Addgene Inc. Cambridge, MA, USA) for 48 hours and followed by treatment of 0.5 mM PA for 2, 4, 8, 10 and 12 hours. Fluorescence signal detection and image capture at 40x magnification were performed using confocal microscope (Carl Zeiss LSM 710; Oberkochen, Germany). The number of GFP and mRFP puncta was quantified manually. The cells were randomly analyzed in three independent experiments.

In some cases, RAW 264.7 macrophages infected with Ad-AFABP or Ad-Luci (50 M.O.I.), for 48 hours or primary macrophages derived from A-FABP KO or WT mice were treated with PA (0.5 mM) or FFA-free BSA supplemented media as control for 8 hours. The endogenous LC3 puncta structure is determined by immunofluorescence staining using primary rabbit anti-LC3 antibody (Cell Signaling Technology, Danvers, Massachusetts, USA) and fluorescence secondary anti-rabbit antibody. Fluorescence signal detection and image capture of the LC3 puncta at 400x magnification were performed using a clinical fluorescence microscope (BX-41 System; Olympus, Hamburg, Germany) with a color digital camera (Olympus Model DP72). Nuclei were stained with 4′,6-diamidino-2-pheylindole (DAPI; Invitrogen).

### Determination of phagocytic activity in macrophages

Phagocytic activities of macrophages were evaluated using phagocytosis assay kit (Cayman Chemical; Ann Arbor, MI, USA) according to the manufacturer manual. In brief, macrophages were plated at a concentration of less than 80% confluence. Cells were incubated with a 1:500 dilution of latex beads-rabbit IgG-FITC complex or no beads at 37 °C for 4 hours. The cells were then incubated with trypan blue quenching solution for 2 minutes and washed with assay buffer for flow cytometry. Cells were then detached by gentle scraping, centrifuged at 400 × g for 5 minutes and resuspended in 2% BSA in PBS. The degree of phagocytosis of latex beads-rabbit IgG-FITC complex in macrophages was monitored by flow cytometry.

### Determination of the M1 or M2 macrophage polarization

Peritoneal macrophages isolated from A-FABP KO mice and WT littermates were treated with LPS (10 ng/ml) and IFNγ (100 ng/ml) or IL4 (10 ng/ml) for 8 hours to induce M1 macrophage polarization or M2 macrophage polarization, respectively. RAW 264.7 cells were transfected with siA-FABP or ScrRNA (Scr) for 48 hours followed by treatment with LPS and IFNγ for 8 hours or infected with either Ad-Luci or Ad-A-FABP for 48 hours followed by treatment with IL4 (10 ng/ml) for 8 hours. The expressions of M1 or M2 macrophage polarization markers were determined by Q-PCR.

### Western blot analysis

Thirty micrograms of protein from the macrophage extract were separated by 8% or 15% SDS-PAGE, transferred onto a polyvinylidene difluoride membrane and probed with various antibodies for unfolded protein response signaling pathway and autophagy markers (Cell signaling Technology Inc.). After incubation with horse radish peroxidase-conjugated IgG secondary antibodies, the proteins were visualized with the enhanced chemiluminescence reagents (GE healthcare, Uppsala, Sweden). The expression level of the target protein was normalized against either the housekeeping β-actin or their relative total non-phosphorylated protein. The blot densities were quantified using the NIH Image J software.

### Quantitative real-time PCR (Q-PCR)

Total RNA was extracted from RAW 264.7 macrophages or peritoneal macrophages using TRIZOL reagent (Invitrogen). One microgram of total RNA was subjected to reverse transcription using the Superscript first-strand cDNA synthesis system (Promega, Madison, WI, USA) according to the manufacturer’s instruction. The relative gene abundance was quantified by real-time PCR using SYBR Green reagent (Qiagen, Venlo, the Netherlands) on an ABI 7000 sequence detection system (Applied Biosystems, Foster, CA, USA). The sequences of the primers are listed in Supplementary Table S1 (see Supplementary Table S1). The relative gene expression was analyzed using the 2^(−ΔΔCT)^ method and normalized against the housekeeping gene β-actin.

### Statistical analysis

All analyses were performed with Statistical Package for Social Sciences version 21.0 (SPSS, Chicago. IL, USA). Data were expressed as mean ± SEM. Statistical significance was determined by One-way ANOVA with Bonferroni correction for multiple comparisons or Student’s t-test. In all statistical comparisons, a *P* value less than 0.05 was considered to indicate statistically significant differences.

## Additional Information

**How to cite this article:** Hoo, R. L. C. *et al*. Adipocyte Fatty Acid Binding Protein Potentiates Toxic Lipids-Induced Endoplasmic Reticulum Stress in Macrophages via Inhibition of Janus Kinase 2-dependent Autophagy. *Sci. Rep.* **7**, 40657; doi: 10.1038/srep40657 (2017).

**Publisher's note:** Springer Nature remains neutral with regard to jurisdictional claims in published maps and institutional affiliations.

## Supplementary Material

Supplementary Tables and Figures
