# Supplementary material for: Adipocyte Fatty Acid Binding Protein Potentiates Toxic Lipids-Induced Endoplasmic Reticulum Stress in Macrophages via Inhibition of Janus Kinase 2-dependent Autophagy
Source: Sci Rep. 2017 Jan 17;7:40657. doi: 10.1038/srep40657 (PMC5240568; doi:10.1038/srep40657)
Supplement: Supplementary Tables and Figures [file srep40657-s1.pdf]

Adipocyte Fatty Acid Binding Protein Potentiates Toxic lipids-Induced Endoplasmic Reticulum Stress in Macrophages via Inhibition of Janus Kinase 2-dependent Autophagy

*Ruby LC Hoo[1,2], Lingling Shu[1,2], Kenneth KY Cheng[1,2,] , Xiaoping Wu[1,2], Boya Liao[1,2] Donghai Wu[3], Zhiguang Zhou[4], Aimin Xu[1,2,5]

[1] State Key Laboratory of Pharmaceutical Biotechnology, [2] Department of Medicine, LKS Faculty of Medicine, The University of Hong Kong, China. [3] Key laboratory of Regenerative Biology, Guangzhou Institute of Biomedicine and Health, Chinese Academy of Sciences, Guangzhou, China. [4] Department of Geriatrics, Second Xiangya Hospital, Central South University, Changsha, Hunan China. [5] Department of Pharmacology and Pharmacy, LKS Faculty of Medicine, The University of Hong Kong, Hong Kong, China.

Email:

1. Ruby LC Hoo: rubyhoo@hku.hk
2. Lingling Shu: shinyshu@hku.hk
3. Kenneth KY Cheng: dorncky@hku.hk
4. Xiaoping Wu: xpwu@hku.hk
5. Boya Liao: liaoboya@hku.hk
6. Donghai Wu: wu_donghai@gibh.ac.cn
7. Zhiguang Zhou: zhouzg@hotmail.com
8. Aimin Xu: amxu@hku.hk

Running Title: A-FABP suppresses JAK2-dependent autophagy

Keywords: Adipocyte fatty acid binding protein, autophagy, endoplasmic reticulum stress, JAK2 signaling, lipotoxicity

***Correspondence author**

Ruby Lai-chong Hoo

State Key Laboratory of Pharmaceutical Biotechnology, Department of Medicine, LKS Faculty of Medicine, The University of Hong Kong. L843, Laboratory block, 21 Sassoon Road, Pokfulam, Hong Kong.

Tel: (852) 3917-9751; Fax: (852) 2816-2095; Email: rubyhoo@hku.hk

Supplementary table S1: Primer sequences for real time quantitative PCR (Q-PCR) and silencing RNA (siRNA)

Mouse A-FABP-F	CCGCAGACGACAGGA
Mouse A-FABP-R	CTCATGCCCTTTCATAAACT
Mouse XBP-1-F	CTCACGGCCTTGTGGTTGA
Mouse XBP-1-R	TCCATTCCCAAGCGTGTTC
Mouse GRP78-F	CCTGTTCCGCTCTACCATGAA
Mouse GRP78-R	TGGAATTCGAGTAGATCCGCC
Mouse CHOP-F	CATACACCACCACACCTGAAAG
Mouse CHOP-R	CCGTTTCCTAGTTCTTCCTTGC
Mouse IL6-F	ATGAAGTTCCTCTCTGCAAGAGACT
Mouse IL6-R	CACTAGGTTTGCCGAGTAGATCTC
Mouse MCP-1-F	CCACTCACCTGCTGCTACTCA
Mouse MCP-1-R	TGGTGATCCTCTTGTAGCTCTCC
Mouse TNFα-F	CCCTCACACTCAGATCATCTTCT
Mouse TNFα-R	GCTACGACGTGGGCTACAG
Mouse Atg7-F	CAGTTTCCAGTCCGTTGAAGTCCT
Mouse Atg7-R	GGGTCCATACATCCACTGAGGTT
Mouse NOS2-F	CCAAGCCCTCACCTACTTCC
Mouse NOS2-R	CTCTGAGGGCTGACACAAGG
Mouse ArgI-F	TGGCTTGCGAGACGTAGAC
Mouse ArgI-R	GCTCAGGTGAATCGGCCTTTT
Mouse MglI-F	TGAGAAAGGCTTTAAGAACTGGG
Mouse MglI-R	GACCACCTGTAGTGATGTGGG
Mouse Mgl2-F	TTAGCCAATGTGCTTAGCTGG
Mouse Mgl2-R	GGCCTCCAATTCTTGAAACCT
Mouse Actin-F	TACCACCATGTACCCAGGCA
Mouse Actin-R	CTCAGGAGGAGCAATGATCTTGAT
Mouse GAPDH-F	ACTCCCACTCTTCCACCTTC
Mouse GAPDH-R	TCTTGCTCAGTGTCCTTGC
Scramble RNA	AAUAUUAUUAAGGCGACAGAG
si-AFABP	GGAUGGAAAUUUGCAUCAA
si-JAK2	GCAAACCAGGAAUGCUCAA
Mouse sXBP-1 F	ACACGCTTGGGAATGGACAC
Mouse sXBP-1 R	CCATGGGAAGATGTTCTGGG

Supplementary Figure S1

Hoo et al

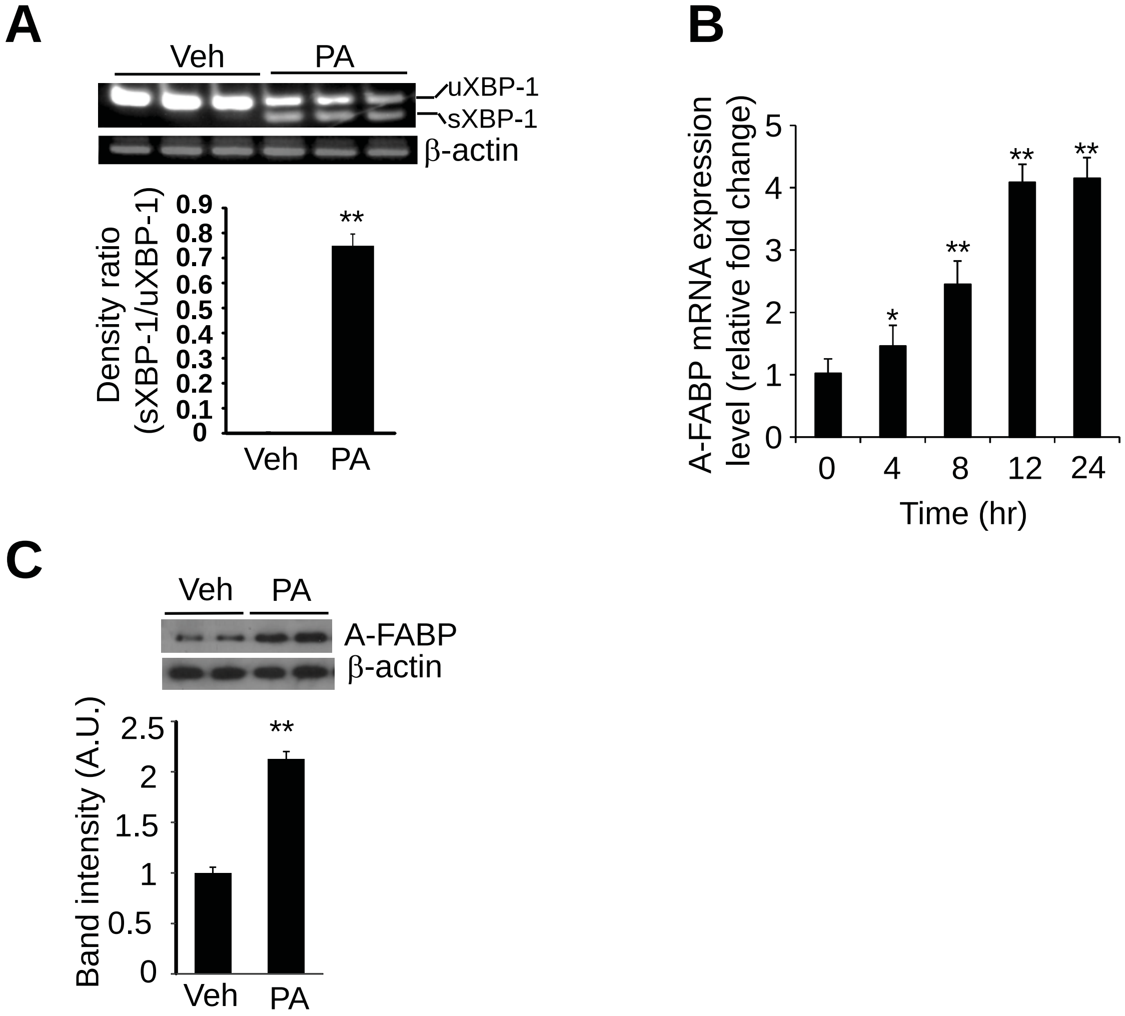

Supplementary Fig. S2

Hoo et al

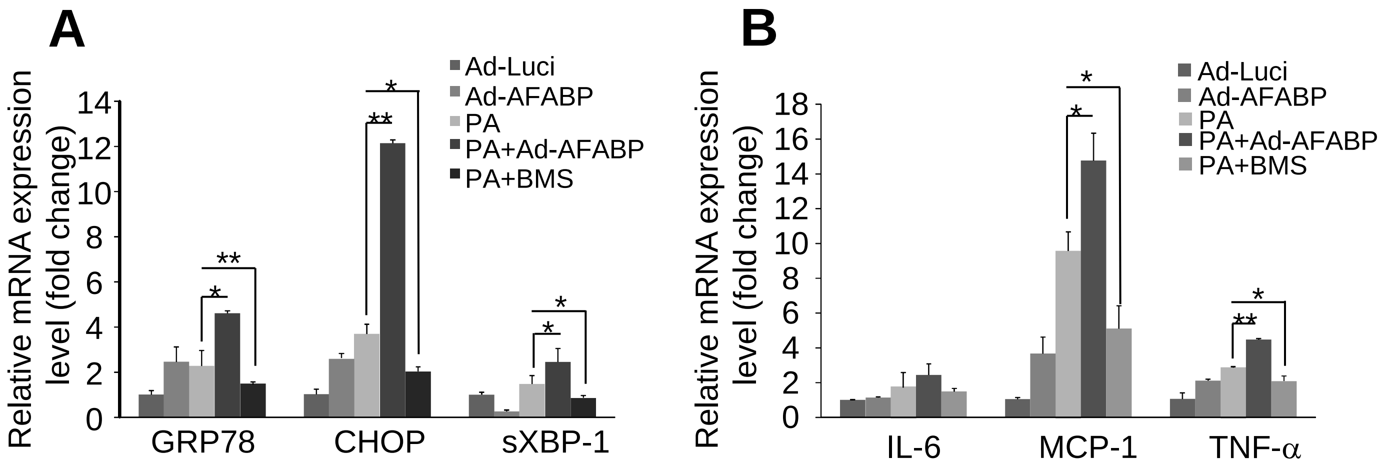

Supplementary Fig. S3

Hoo et al

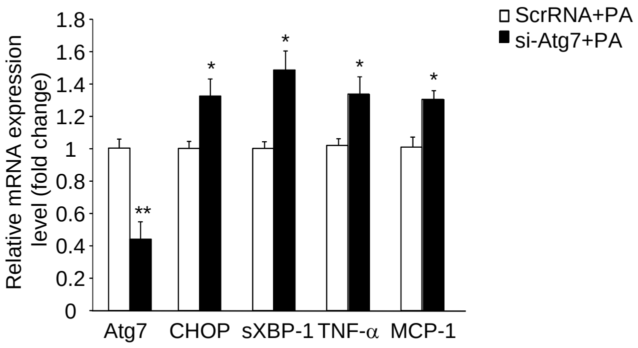

Figure legend for supplementary fig

Supplementary Fig. S1: Treatment of palmitic acid induces ER stress and expression of A-FABP.

(A) RAW264.7 macrophages were treated with either vehicle (Veh) or PA (0.5mM) for 8 hours followed by RNA extraction and reverse transcription. Complementary DNA (cDNA) was subjected to PCR using primers specific to XBP-1 for the detection of unspliced XBP-1 (uXBP-1) and spliced XBP-1 (sXBP-1). The density ratio of sXBP-1/ uXBP-1 was shown in the lower panel. RAW264.7 macrophages were treated with either Veh or PA for 8 hours or as indicated. (B) The mRNA abundance and (C) protein expression levels of A-FABP were measured by real-time PCR and Western blot analysis, respectively. The relative expression level of protein was normalized with β-actin expression and the densitometric quantification of the immunoblot was shown in the lower panel while mRNA abundance was normalized with the house-keeping gene β-actin. Values are expressed as means± S.E.M. *P<0.05; **P <0.01; n=6.

Supplementary Fig. S2: A-FABP enhances PA-induced mRNA expression of ER stress markers and inflammatory cytokines in macrophages. RAW264.7 macrophages infected with either Ad-Luci or Ad-AFABP for 48 hours, or pretreated with BMS (25 μM) for 24 hours were treated with Veh or PA for 8 hours. The mRNA abundance of (A) ER stress markers GRP78, CHOP and spliced XBP-1 (sXBP-1) and (B) inflammatory cytokines (IL-6, MCP-1 and TNF-α) was measured by Q-PCR. Values are expressed as means ± S.E.M. and were normalized with the house-keeping gene β-actin. *P< 0.05; **P< 0.01, n=6.

Supplementary Fig. S3: Inhibition of autophagy in macrophages by knocking down the autophagy related protein (Atg)-7 promotes PA-induced ER stress and inflammatory cytokine production. RAW264.7 macrophages transfected with scramble RNA (ScrRNA) or silencing RNA of Atg7 (si-Atg7) for 48 hours were treated with PA for 8 hours. The mRNA abundance of Atg7, ER stress markers (CHOP and sXBP-1) and inflammatory cytokines (MCP-1 and TNF-α) was measured by Q-PCR. Values are expressed as means±S.E.M. and were normalized with the house-keeping gene β-actin. *P< 0.05; **P< 0.01, n=6.
